# Enhancing Emission and Stability in Na-Doped Cs_3_Cu_2_I_5_ Nanocrystals

**DOI:** 10.3390/nano14131118

**Published:** 2024-06-28

**Authors:** Na Guo, Lili Liu, Guilong Cao, Shurui Xing, Jingying Liang, Jianjun Chen, Zuojun Tan, Yuequn Shang, Hongwei Lei

**Affiliations:** 1 College of Engineering, Huazhong Agricultural University, Wuhan 430070, China; guona136@webmail.hzau.edu.cn (N.G.); liulili53@163.com (L.L.); cao123@webmail.hzau.edu.cn (G.C.); xsr@webmail.hzau.edu.cn (S.X.); liangjingying@webmail.hzau.edu.cn (J.L.); chenjianjun@mail.hzau.edu (J.C.); 2 Department of Physics, Chemistry and Biology (IFM), Linköping University, SE-58183 Linköping, Sweden

**Keywords:** Cs_3_Cu_2_I_5_ NCs, NaI-assisted synthesis, Na-doping, stability, deep-blue-LEDs

## Abstract

Lead-free Cs_3_Cu_2_I_5_ metal halides have garnered significant attention recently due to their non-toxic properties and deep-blue emission. However, their relatively low photoluminescence quantum efficiency and poor stability have limited their applications. In this work, sodium iodide (NaI) is used to facilitate the synthesis of Cs_3_Cu_2_I_5_ nanocrystals (NCs), demonstrating improved photoluminescence intensity, photoluminescence quantum yield, and stability. Systematic optoelectronic characterizations confirm that Na^+^ is successfully incorporated into the Cs_3_Cu_2_I_5_ lattice without altering its crystal structure. The improved Photoluminescence Quantum Yield (PLQY) and stability are attributed to the strengthened chemical bonding, which effectively suppresses vacancy defects in the lattice. Additionally, light-emitting diodes (LEDs) based on 10% NaI-doped Cs_3_Cu_2_I_5_ NCs were assembled, emitting vibrant blue light with a maximum radiant intensity of 82 lux and Commission Internationale de l’Eclairage (CIE) chromaticity coordinates of (0.15, 0.1). This work opens new possibilities for commercial lighting display applications.

## 1. Introduction

Novel organic–inorganic hybrid perovskite materials for LEDs have garnered significant scientific interest due to their low cost, high efficiency, and straightforward manufacturing processes [1,2,3,4,5]. The highest external quantum efficiency (EQE) reported has surpassed 20%, comparable to that of organic and colloidal quantum dot LEDs [6]. However, the toxicity of lead-based perovskites and the lack of high-efficiency perovskite deep-blue LEDs pose challenges to their widespread adoption [7,8]. Consequently, developing non-toxic, high-performance, stable deep-blue emitting materials is crucial [9,10,11].

Among various lead-free perovskites, copper halide perovskites like Cs_3_Cu_2_I_5_ have shown promise with robust self-trapped exciton (STE) emissions [12,13,14]. Since 2019, further insights into the STE mechanisms of Cs_3_Cu_2_I_5_ have been revealed by Cheng and colleagues, attracting significant attention for its pure-blue emissions [15]. Investigations by Wang et al. [16] and Zhao et al. [17] demonstrated high-efficiency deep-blue and cold-white UV-pumped LEDs using Cs_3_Cu_2_I_5_, achieving high color rendering indices (CRI) up to 90. However, Cs_3_Cu_2_I_5_ is susceptible to phase transitions under high radiation and in polar solvents, which impairs its emission performance [13,18,19]. Moreover, its photoluminescence quantum yield (PLQY) lags that of lead-based materials, making enhancements in PLQY and stability vital for its application [20,21,22,23].

Metal ion-mediated synthesis has proven effective in modulating the optoelectronic properties of both lead-based and lead-free perovskites, particularly with small-radius alkali metal cations [24,25,26,27,28,29]. For instance, Na^+^-doping in CsPbBr_3_ and Cs_2_AgInCl_6_ significantly improved PLQY, reducing nonradiative recombination and effectively controlling self-trapped excitons (STEs) [6,30]. These advancements underscore the potential of a tailored doping strategy for Cs_3_Cu_2_I_5_ to boost its emission performance and stability.

In this study, we employ a Na-doping strategy in Cs_3_Cu_2_I_5_ nanocrystals to enhance both PLQY and stability. Our systematic characterizations show successful interstitial doping of Na^+^, which notably improves the PL intensity (100% enhancement), the PLQY (10% enhancement), and the stability (30% enhancement) by minimizing defects and inhibiting ion migration. Moreover, stable light-emitting diodes (LEDs) have been fabricated, and blue light which is close to the standard blue spectrum has been achieved.

## 2. Results and Discussion

### 2.1. Structural and Morphological Characteristics

**Figure 1:**
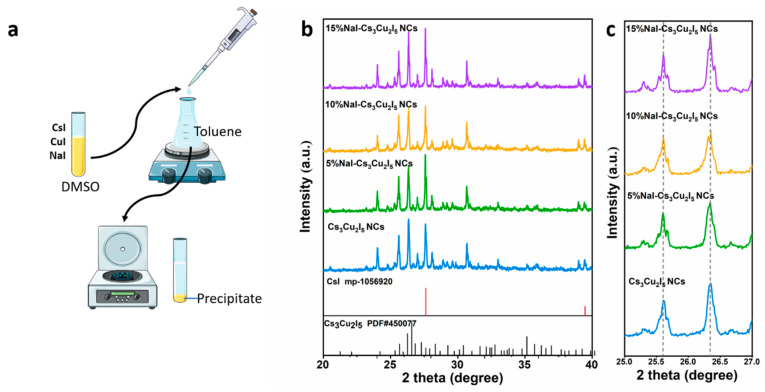
(**a**) Systematic illustration of the synthesis method. (**b**) XRD patterns of *x*NaI-Cs_3_Cu_2_I_5_ (*x* = 0, 5%, 10%, 15%) powders. (**c**) Detailed XRD patterns of *x*NaI-Cs_3_Cu_2_I_5_ (*x* = 0, 5%, 10%, 15%) in a specific range.

A series of xNaI-Cs_3_Cu_2_I_5_ (x = 0, 5%, 10%, 15%) nanocrystals (NCs) were synthesized using an anti-solvent method, as illustrated in Figure 1a. Note that the ratio of CsI and CuI in the precursor is optimized at 2:1 to ensure pure phase and high PL intensity, as shown and explained in the supporting information (Figure S1). In addition, for a better clarity, the content of Na in the final product is calculated by the quantitative X-ray photoelectron spectroscopy (XPS) analysis as 6.32% for the 10% NaI-doped precursor. The phase and structure of the samples were characterized by X-ray diffraction (XRD). As depicted in Figure 1b, the XRD patterns confirm that all NCs crystallize in the orthorhombic system with a *Pnma* space group. The diffraction peaks closely match the standard PDF#450077 (JCPDS) [31,32], confirming the crystallinity of the samples and indicating that Na^+^ doping does not alter the Cs_3_Cu_2_I_5_ crystal lattice structure. The primary peaks at 13.1°, 15.1°, 23.9°, 25.6°, 26.3°, 28.2°, and 30.6° correspond to the (111), (002), (122), (312), (222), (131) and (313) planes of Cs_3_Cu_2_I_5_, respectively. The Rietveld refinement of the XRD pattern is shown in Figure S2, reconfirming the space group and revealing the lattice parameters of the Na-dope Cs_3_Cu_2_I_5_. As shown in Figure 1c, the peak at 25.6° remains unchanged with increasing Na^+^ concentration, indicating stable lattice integration. Additionally, there are no NaI-related diffraction peaks observed at different doping concentrations, further confirming the successful incorporation of Na^+^ into the Cs_3_Cu_2_I_5_ lattice. A detailed analysis of the XRD patterns also reveals additional peaks at 27.6° and 39.5°, which are attributable to the (110) and (200) planes of the excess CsI.

**Figure 2:**
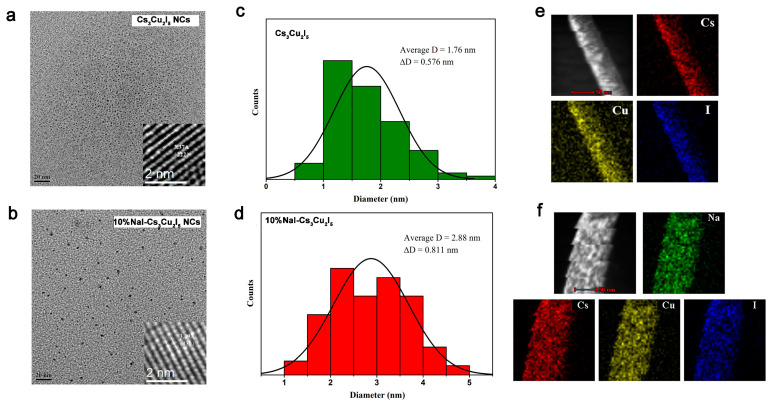
TEM and HRTEM image of Cs_3_Cu_2_I_5_ NCs (**a**) and 10%NaI-Cs_3_Cu_2_I_5_ NCs (**b**). Particle size distribution histogram of Cs_3_Cu_2_I_5_ NCs (**c**) and 10%NaI-Cs_3_Cu_2_I_5_ NCs (**d**). Elemental mapping images of Cs_3_Cu_2_I_5_ NCs (**e**) and 10%NaI-Cs_3_Cu_2_I_5_ NCs (**f**).

The morphology and fine structure of the obtained nanocrystals were further analyzed using high-resolution transmission electron microscopy (HRTEM). Compared to the control sample, Na^+^ doping can significantly improve the crystallinity of the nanocrystals, resulting in larger crystal sizes (Figure 2a,b). This result is in consistent with the analysis of the XRD pattern, as shown in Table S1. The inserted HRTEM images of Cs_3_Cu_2_I_5_ and 10%NaI-Cs_3_Cu_2_I_5_ NCs were analyzed to characterize the fine lattice structure. For Cs_3_Cu_2_I_5_ NCs, the lattice spacing is 3.37 Å, consistent with the lattice plane of (222). 10%NaI-Cs_3_Cu_2_I_5_ shows a lattice spacing of 3.29 Å, aligning with the lattice plane of (130), reaffirming that Na^+^ doping does not alter the crystal structure, corroborating the XRD pattern. Both samples display distinct and regular lattice fingers, indicating high crystallinity (Figure S3). In addition, as shown in Figure 2c,d, the average diameter of Cs_3_Cu_2_I_5_ is 1.76 nm with a size deviation of ±0.576 nm, while the average diameter of 10%NaI-Cs_3_Cu_2_I_5_ is 2.88 nm with a size deviation of ±0.811 nm, increased by 63.6%, which also confirms the formation of higher quality Cs_3_Cu_2_I_5_ NCs. Moreover, Figure 2e,f showcase the high-resolution elemental mapping images of Cs_3_Cu_2_I_5_ and 10%NaI-Cs_3_Cu_2_I_5_ NCs, further confirming the uniform doping and distribution of Na^+^ and the formation of high-quality nanocrystals. 

### 2.2. Optical Properties

**Figure 3:**
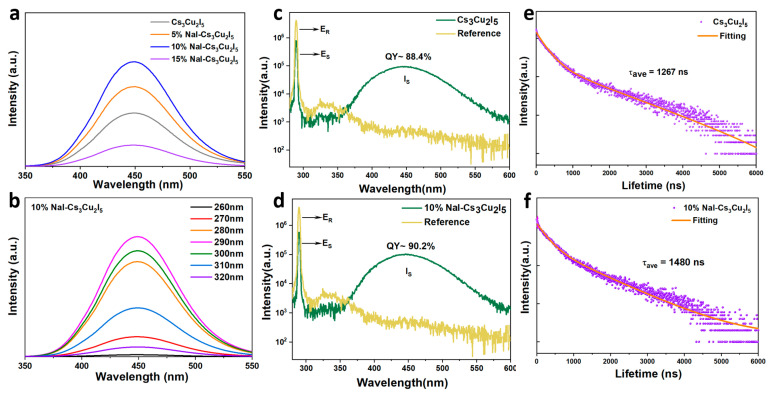
(**a**) The PL spectra of Cs_3_Cu_2_I_5_@xNaI (x = 0, 5%, 10%, 15%) NCs; (**b**) the PL emission spectra of Cs_3_Cu_2_I_5_@10%NaI NCs at different excitation wavelengths; PLQY of Cs_3_Cu_2_I_5_ NCs with (**c**) and without (**d**) Na^+^ doping; PL lifetime of Cs_3_Cu_2_I_5_ NCs with (**e**) and without (**f**) Na^+^ doping.

Figure 3a shows the PL intensity of Cs_3_Cu_2_I_5_ nanocrystals doped with different concentrations of NaI. Notably, as the doping concentration increases, the PL intensity first increases and then decreases. The maximum PL intensity is achieved when the NaI doping concentration reaches 10%, which shows a 100% improvement compared with the control samples. Figure 3b displays the excitation-wavelength-dependent PL characteristics of the 10%NaI-Cs_3_Cu_2_I_5_ NCs. It is evident that the PL spectra at different excitation wavelengths maintain the same features, showing the same peak positions for the undoped sample. This consistency indicates that NaI doping does not alter the emission mechanism.

Photoluminescence quantum yield (PLQY) is an important parameter describing emission performance, defined as the ratio of the number of emitted photons to the number of absorbed photons. The specific formula for calculating is as follows:

(1) PLQY=∫IS∫(ER-ES)×100%

where I_S_ represents the integrated area of the PL spectrum, and E_R_ and E_S_ correspond to the integrated areas of the excitation spectrum without and with the sample, respectively. Figure 3c,d show the PLQY of Cs_3_Cu_2_I_5_ NCs without and with NaI doping. The results prove that NaI doping can enhance the emission performance, yielding a PLQY value of over 90% which is among the high-level PLQY values in the literature reports (Table S2).

To further understand the impact of NaI doping on the excited state dynamics, time-resolved photoluminescence spectra were obtained of Cs_3_Cu_2_I_5_ NCs and 10% NaI-Cs_3_Cu_2_I_5_ NCs under 290 nm pulsed laser excitation, as shown in Figure 3e,f. The obtained data were fitted by bi-exponential mode (Equation (2)), with the two components attributed to radiative recombination (longer lifetime τ_2_) and trap-mediated recombination (shorter lifetime τ_1_). Here, A(t) signifies the PL intensity at time t. The parameters A_1_ and A_2_ are utilized in the fitting process to represent the amplitudes of two exponential decay components. Meanwhile, τ_1_ and τ_2_ are employed to denote the lifetimes associated with these two exponential decay terms.

(2) At=A1exp−tτ1+A2exp−tτ2

For the Cs_3_Cu_2_I_5_ and 10%NaI-Cs_3_Cu_2_I_5_ samples, their average lifetimes have been determined to be 1267 ns and 1480 ns, and the corresponding results are shown in Table S3. The calculated τ_1_ is improved to 1466 ns for the doped sample, implying that the trap or defect concentration is reduced by the Na^+^ dopant. The fitted value of calculated τ_2_ is also increased, demonstrating that NaI effectively decreases the potential non-radiative recombination. Therefore, Na^+^ doping shows enhanced photoluminescence performance. This can be partly attributed to the enhanced crystallinity of Na-doped samples, and more underlying mechanisms will be discussed in the following text.

### 2.3. Stability

**Figure 4:**
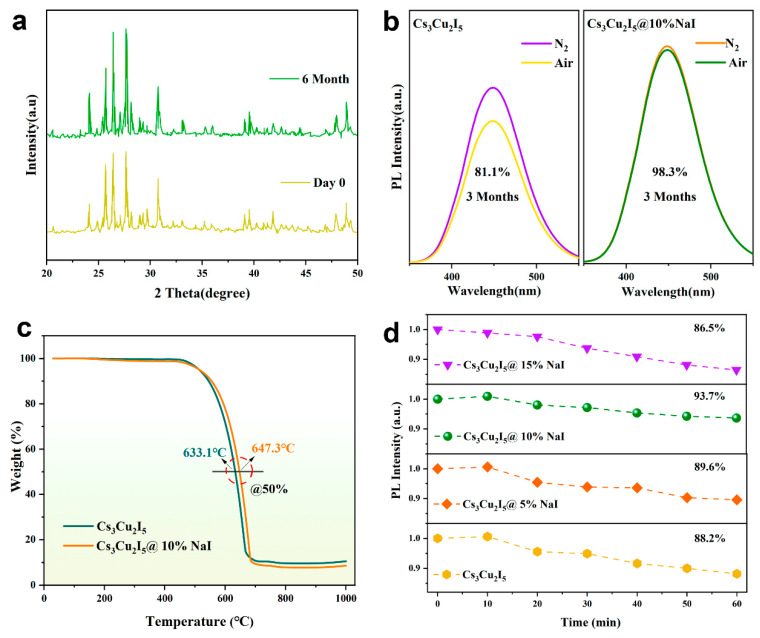
(**a**) XRD patterns of the NCs before and after 6 months of aging. (**b**) Comparison of the stability in nitrogen and air for three months. (**c**) Thermogravimetric of doped and undoped NCs; (**d**) The PL spectra of *x*NaI-Cs_3_Cu_2_I_5_ (*x* = 0, 5%, 10%, 15%) under 60 min of UV irradiation.

We conducted comprehensive environmental stability studies to evaluate the suitability of 10%NaI-Cs_3_Cu_2_I_5_ NCs for LED applications, including air exposure, temperature, moisture, and UV irradiation. In the initial experiment, the nanocrystals were exposed to ambient air for six months, and their X-ray diffraction (XRD) patterns demonstrate exceptional phase stability, as shown in Figure 4a, indicating that even after prolonged exposure to external conditions, their crystal structure does not show significant changes. Meanwhile, tracking the photoluminescence performance further proves the significant role of NaI doping in enhancing air stability, as shown in Figure 4b. Compared to the undoped samples which maintain 81.1% of their PL intensity after three months of aging in air, the 10%NaI-Cs_3_Cu_2_I_5_ NCs did not show significant PL intensity attenuation. 

We used thermogravimetric analysis to assess the thermal stability of Cs_3_Cu_2_I_5_ and 10%NaI-Cs_3_Cu_2_I_5_ NCs, with the results shown in Figure 4c. It was found that the decomposition temperature of 10%NaI-Cs_3_Cu_2_I_5_ NCs is higher than undoped samples, which may be related to the formation of high-quality crystals induced by doping. Furthermore, we investigated photoluminescence changes under prolonged 254 nm UV lamp irradiation to study the stability of these nanocrystals in more detail. The results, shown in Figure 4d, demonstrate that the 10% doped nanocrystals exhibit better stability than the undoped nanocrystals (88.2%), with photoluminescence intensity retaining 93.7% of the initial value after 60 min of UV irradiation. The accelerated humidity stability tests by dispersing the NCs into a mixture of isopropanol and water were also conducted, revealing that 10%NaI-Cs_3_Cu_2_I_5_ NCs have better humidity stability, as shown in Figure S4.

### 2.4. Mechanisms of the Enhanced Photoluminescence Performance and Stability

**Figure 5:**
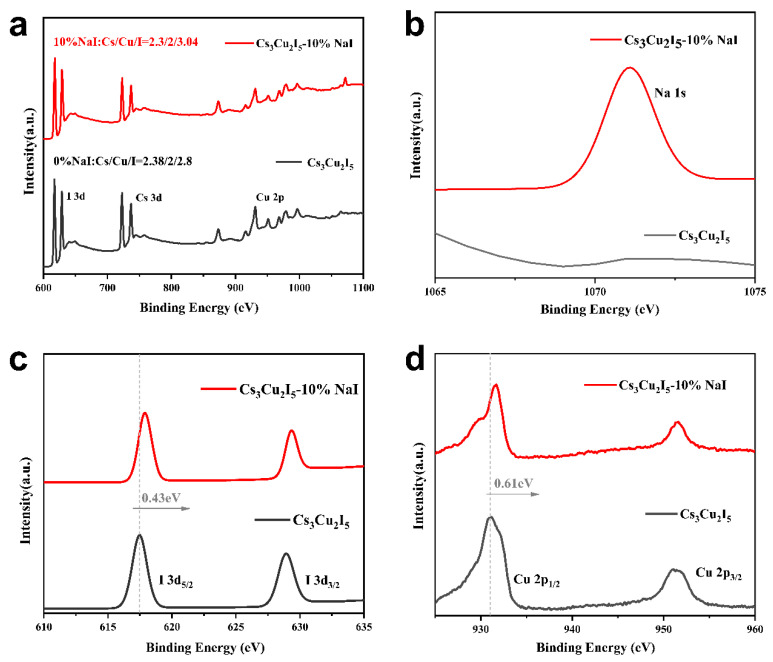
XPS spectra of Cs_3_Cu_2_I_5_ and 10% NaI-Cs_3_Cu_2_I_5_ (**a**) whole survey spectrum, (**b**) Na 1s spectrum, (**c**) I 3d spectrum, and (**d**) Cu 2p spectrum.

To understand the mechanisms underlying the improved PL, PLQY and stability in NaI-doped Cs_3_Cu_2_I_5_ NCs, X-ray Photoelectron Spectroscopy (XPS) measurements were performed to examine the chemical state and chemical bonds after NaI doping. In Figure 5a, the survey spectra of the 10% NaI-doped and undoped samples are presented. Through semi-quantitative analysis of different elements, we can observe a significant change in the Cu-I ratio in the NCs before and after doping, shifting from 2:2.8 in the undoped samples to 2:3.04. This intuitively indicates that NaI doping has significantly altered the potential path of crystal growth, effectively suppressing the formation of I vacancies. Additionally, a notable Na 1s signal appears at 1071 eV (Figure 5b), indicating that Na^+^ has been successfully integrated into the NCs. Furthermore, the fine spectra of I 3d and Cu 2p are shown in Figure 5c,d, respectively. After Na^+^ doping, the peak positions of I 3d and Cu 2p have shifted towards higher binding energy by 0.43 eV and 0.61 eV, respectively. This change suggests that the chemical bonding between Cu^+^ and I^−^ in Cs_3_Cu_2_I_5_ NC has strengthened, which helps suppress I vacancy defects and enhances the PL, PLQY, and stability.

### 2.5. Demonstration of LEDs

**Figure 6:**
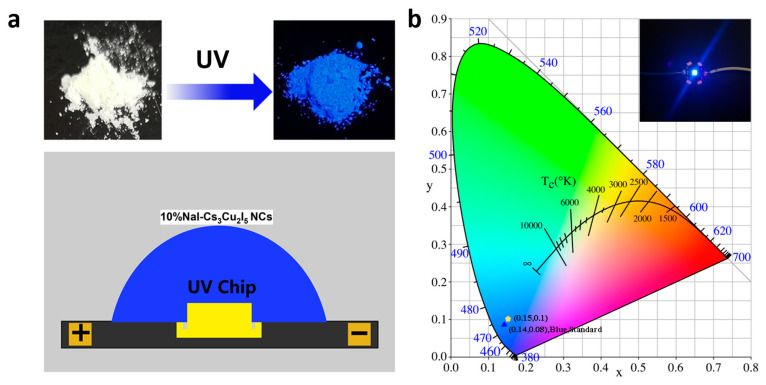
(**a**) Systematic illumination of the down-conversion LED. (**b**) CIE coordinate diagram of Na^+^ doped Cs_3_Cu_2_I_5_ NCs-based LEDs. The inset is a photograph of a prepared blue-emitting LED based on Na^+^-doped Cs_3_Cu_2_I_5_ NCs.

The high-quality Cs_3_Cu_2_I_5_ nanocrystals synthesized via Na^+^ doping, with their exceptional environmental stability, significantly improved PL, PLQY, and standard blue emission capability, are promising candidates for optoelectronic display applications. Here, we preliminarily demonstrate their application in down-conversion lighting. As shown in Figure 6a, down-conversion LED devices were fabricated by combining UV LED chips with our nanocrystal powders. Under a forward bias current of 15 mA, the LED emits vibrant blue light with a maximum achievable radiant intensity of 82 lux and exhibits CIE chromaticity coordinates of (0.15, 0.1), as shown in Figure 6b, meeting the requirements of standard blue display specified by National Television Standards Committee (NTSC) [33,34]. To further improve the optical and photoluminescent properties of Cs_3_Cu_2_I_5_ for practical applications, we should focus on fabricating high-quality films or crystals with stable local structures, reducing the recombination centers in the materials, introducing additional light-emitting centers such as rare-earth elements, and modifying the compositions such as manipulating the ratio of CsCu_2_I_3_ and Cs_3_Cu_2_I_5_ to obtain white-emitting LEDs. 

## 3. Conclusions

This study delves into the impact of alkali metal Na^+^ doping on the PL intensity, PLQY and stability of Cs_3_Cu_2_I_5_ nanocrystals. Detailed structural characterization fully proved that Na^+^ was successfully incorporated into the Cs_3_Cu_2_I_5_ crystals and significantly improved the crystallinity. Further photoluminescence performance characterization showed that as the Na^+^ doping concentration increased, the PL intensity exhibited a trend of first rising and then falling, reaching the maximum PL at 10% Na^+^ doping concentration with a high PLQY of over 90%. Moreover, the average PL lifetime was increased by 16.8% with 10% Na^+^ doping. Various environmental stability tests showed that the doped Cs_3_Cu_2_I_5_ nanocrystals had lower sensitivity to air exposure, temperature changes, and UV light, processing excellent stability. The XPS results explained in depth the reasons for the improved PL intensity, PLQY, and stability of doped Cs_3_Cu_2_I_5_ nanocrystals, highlighting the stronger chemical bonding between Cu^+^ and I^-^ in the Cs_3_Cu_2_I_5_ nanocrystals, significantly suppressing the formation of I vacancy defects. Finally, we demonstrated the utilization of doped Cs_3_Cu_2_I_5_ nanocrystals in down-conversion LEDs, showing high-performance blue emission that meets NTSC standard displays, which opens more possibilities for potential commercial lighting display applications. 

## 4. Experimental Section

### 4.1. Materials and Chemicals

Cesium Iodide (CsI, 99.999%) and Sodium Iodide (NaI, 99.99%) were purchased from Aladdin. Copper(I) Iodide (CuI, 99%) was purchased from Macklin. Dimethyl Sulfoxide and Poly(methyl methacrylate) (PMMA, average Mw ~350,000) were purchased from Sigma-Aldrich. Toluene and Isopropanol were purchased from Sinopharm. All the chemicals were used as received without further purification.

### 4.2. Synthesis of Na-Doped Cs_3_Cu_2_I_5_ Nanocrystals

The synthesis of Cs_3_Cu_2_I_5_ nanocrystals follows the widely used anti-solvent method at room temperature. In brief, 0.6 mmol of CsI and 0.3 mmol of CuI were dissolved in 7.5 mL of DMSO as the precursor in the glove box. Regarding the Na^+^-doped Cs_3_Cu_2_I_5_ nanocrystals synthesis, a series of precursor solutions for *x*NaI-Cs_3_Cu_2_I_5_ (*x* = 0, 5%, 10%, 15%) nanocrystals were prepared by co-solving NaI with molar ratios of 0, 5%, 10%, and 15% in the above precursor. As an example, 10% NaI-Cs_3_Cu_2_I_5_, 0.6 mmol of CsI, 0.3 mmol of CuI, and 0.015 mmol of NaI were diluted in 7.5 mL of DMSO, and sonicated until completely dissolved to form precursor solutions. Subsequently, 200 μL of the precursor solution was injected into 5 mL toluene under vigorous stirring in the air. Then, the solution was centrifuged for 5 min at 8000 rpm. After centrifugation, the supernatant was discarded, and the precipitate was redispersed in isopropanol to form a long-term stable colloidal solution for subsequent characterization. 

### 4.3. Fabrication of LEDs

The centrifuged precipitate is dried in a vacuum drying oven at 60 ℃ and ground with an agate mortar to obtain a fine powder. Na-doped Cs_3_Cu_2_I_5_ NCs powder, as blue light emitters, was mixed with PMMA in 1 mL of toluene at a weight ratio of 1:20 and stirred well. Finally, the paste-like mixture was dropped on a 290 nm UV LED chip and dried under vacuum at 60 °C for 1 h to prepare NCs-PMMA composite LEDs. 

### 4.4. Characterizations

XRD was performed using a Bruker D8 X-ray diffractometer with a radiation source from the Cu target (Kɑ = 1.5406 Å). An FEI Tecnai G2 F20 transmission electron microscope (TEM) was used to assess the fine morphology and lattice structure; energy dispersive X-ray spectroscopy (EDXS) surface scan was used to obtain the elemental distribution (mapping) and EDX semi-quantitative data. XPS was conducted on a Thermo Scientific ESCALAB Xi+ instrument. Thermogravimetric analysis (TGA) was measured using a Discovery TGA 550 with a heating rate of 5 °C/min under a nitrogen atmosphere. The PL excitation (PLE) and PL spectra were measured using a Shimadzu RF-6000 fluorescence spectrophotometer. The measurement of photoluminescence quantum yield was performed using an Edinburgh FLS1000 with an integrating sphere, and the photoluminescence lifetime was obtained using an Edinburgh FLS1000 at the specified wavelengths with a microsecond pulsed laser, the measurement of absorption spectra was performed on a UV–Vis 1800 UV–Vis absorption spectrometer. 

## Data Availability

The data that support the findings of this study are available on request from the corresponding author.

## Supplementary Materials

The following supporting information can be downloaded at: https://www.mdpi.com/article/10.3390/nano14131118/s1, Figure S1: XRD patterns (a) and photoluminescence patterns (b) of Cs3Cu2I5 NCs prepared from different raw material ratios. The black asterisk represents a peak of Cs3Cu2I5, while the red asterisk indicates two principal peaks of CsCu2I3; Figure S2: The Rietveld refinement of the XRD pattern for Na-dope Cs3Cu2I5; Figure S3: Diffraction pattern of Cs3Cu2I5 NCs (a); of Cs3Cu2I5@10%NaI NCs (b); Figure S4: Water stability of Cs3Cu2I5@xNaI (x = 0, 5%, 10%, 15%) NCs; Table S1: Analysis of XRD parameters for Cs3Cu2I5@xNaI (x = 0, 5%, 10%, 15%); Table S2: Summary of NCs properties; Table S3: PL lifetime of NCs (λex = 290 nm, λem = 450 nm).
