# Supplementary material for: Enhancing Emission and Stability in Na-Doped Cs3Cu2I5 Nanocrystals
Source: Nanomaterials (Basel). 2024 Jun 28;14(13):1118. doi: 10.3390/nano14131118 (PMC11243144; doi:10.3390/nano14131118)
Supplement: Supplementary file 1 [file nanomaterials-14-01118-s001.zip › nanomaterials-3065064-supplementary.pdf]

Supplementary Information

Enhancing Emission and Stability in Na-Doped $Cs_3Cu_2I_5$ Nanocrystals

Na Guo [1], Lili Liu [1], Guilong Cao [1], Shurui Xing [1], Jingying Liang [1], Jianjun Chen [1], Zuojun Tan [1,*], Yuequn Shang [2,*] and Hongwei Lei [1,*]

1 College of Engineering, Huazhong Agricultural University, Wuhan 430070, China; guona136@webmail.hzau.edu.cn (N.G.); liulili53@163.com (L.L.); cao123@webmail.hzau.edu.cn (G.C.); xsr@webmail.hzau.edu.cn (S.X.); liangjingying@webmail.hzau.edu.cn (J.L.); chenjianjun@mail.hzau.edu (J.C.)

2 Department of Physics, Chemistry and Biology (IFM), Linköping University, SE-58183 Linköping, Sweden

* Correspondence: tzj@mail.hzau.edu.cn (Z.T.); yuequn.shang@liu.se (Y.S.); leihw@mail.hzau.edu.cn (H.L.)

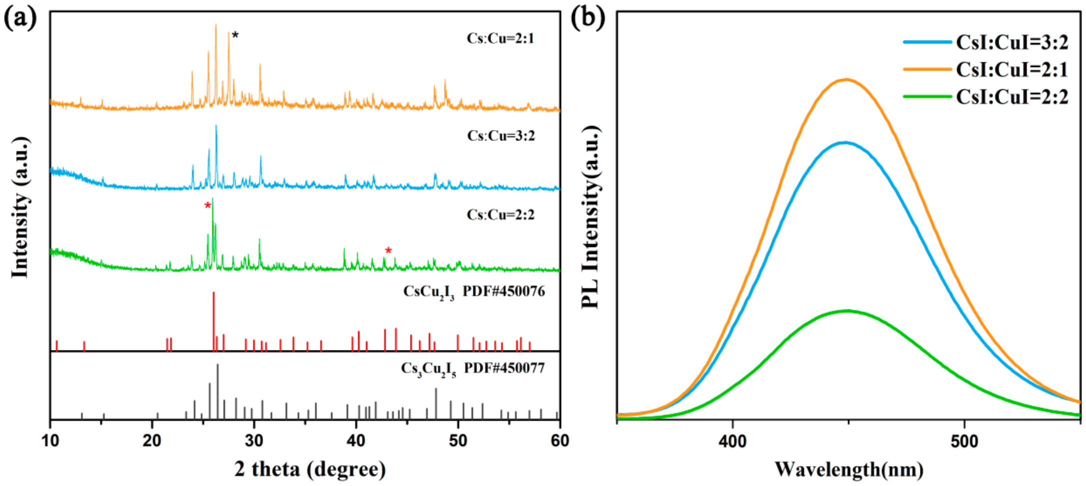

Figure S1. XRD patterns (**a**) and photoluminescence patterns (**b**) of $Cs_3Cu_2I_5$ NCs prepared from different raw material ratios. The black asterisk represents a peak of $Cs_3Cu_2I_5$, while the red asterisk indicates two principal peaks of $CsCu_2I_3$

The XRD results show that only two products, $CsCu_2I_3$ and $Cs_3Cu_2I_5$, are synthesized when different ratios of CsI and CuI are added, as shown in Figure S1a. When CsI: CuI=2:2, two phases of $CsCu_2I_3$ and $Cs_3Cu_2I_5$ were generated, which was not conducive to the preparation of blue LEDs due to the yellow light emission of $CsCu_2I_3$. When CsI: CuI=3:2, the XRD pattern showed that the characteristic peak of $CsCu_2I_3$ disappeared, indicating that the addition of excess CsI was beneficial to the suppression of $CsCu_2I_3$ generation. Gradually increasing the amount of CsI, when the ratio of CsI: CuI reached 2:1, the characteristic peak of $CsCu_2I_3$ also did not appear, indicating that the pure phase of $Cs_3Cu_2I_5$ was synthesized, but the characteristic peak of CsI appeared at 27.6°, corresponding to the (110) crystal plane of CsI, which was caused by the addition of excess CsI, but CsI did not emit light, nor did it affect the luminescence of $Cs_3Cu_2I_5$ NCs. Meanwhile, comparing the PL intensity of the NCs synthesized with different precursor ratios, as shown in Figure S1b, it can be found that the strongest PL intensity is obtained when CsI:CuI=2:1, because the excess CsI utilizes all the CuI as the luminescent center[1]. Therefore, to pursue pure blue light and the best luminescence, CsI: CuI=2: 1 was chosen as the best precursor ratio in this paper.

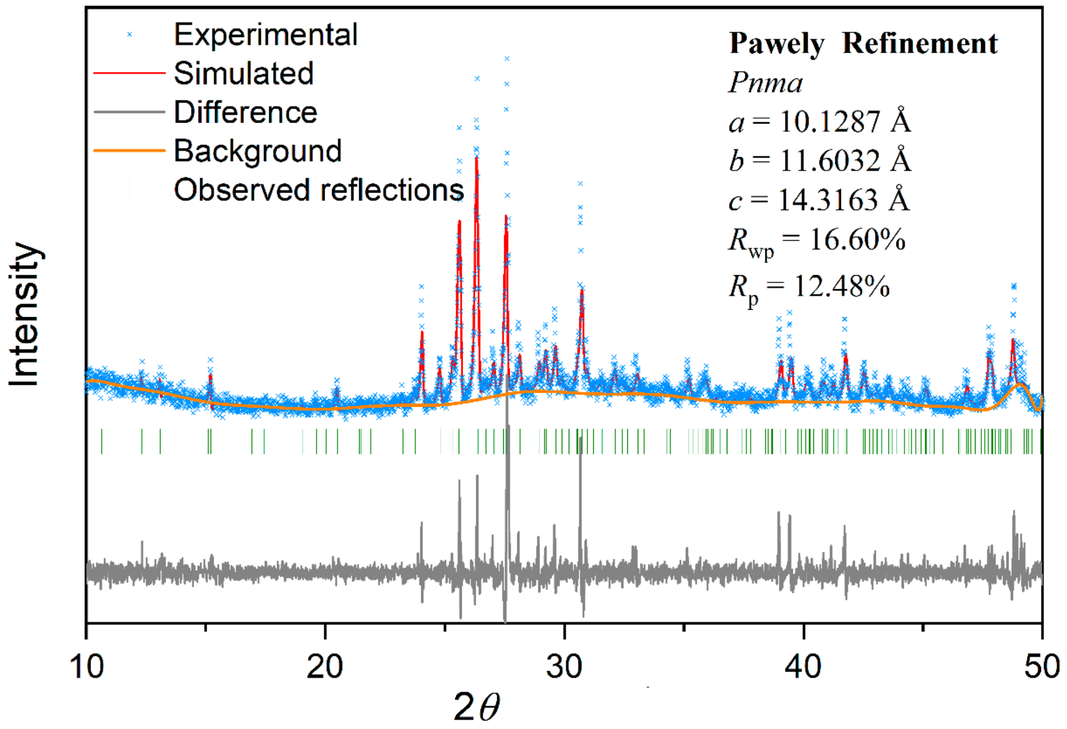

Figure S2. The Rietveld refinement of the XRD pattern for Na-dope $Cs_3Cu_2I_5$.

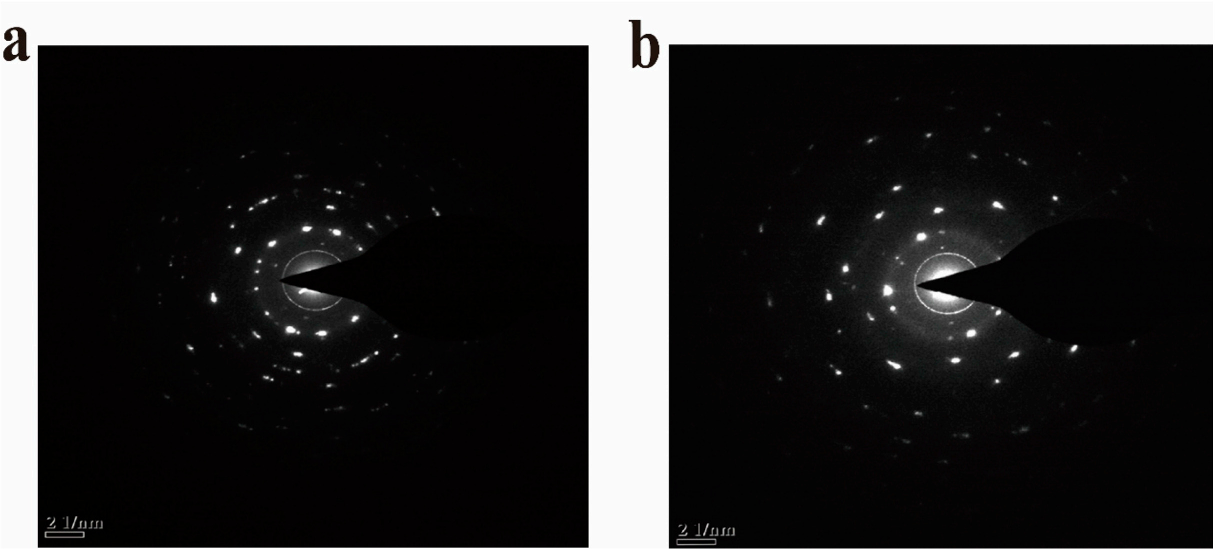

Figure S3. Diffraction pattern of $Cs_3Cu_2I_5$ NCs (**a**); of $Cs_3Cu_2I_5$@10%NaI NCs (**b**).

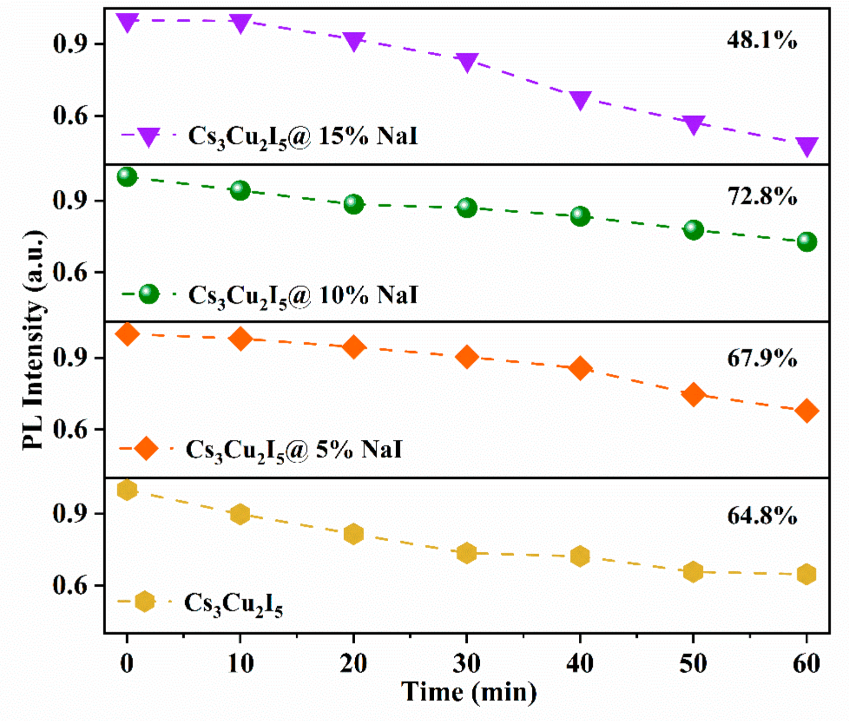

Figure S4. Water stability of $Cs_3Cu_2I_5$@xNaI (x=0, 5%, 10%, 15%) NCs.

To investigate the impact of moisture on luminescence properties, the nanocrystals were lastly dispersed into a 3:1 volume ratio mixture of isopropanol and water, and their PL intensity was recorded every ten minutes. The experimental results, which are displayed in Figure S4, demonstrate that after an hour of immersion in the aqueous solution, there is a significant decrease in emission intensity.

Table S1. Analysis of XRD parameters for Cs3Cu2I5@xNaI (x=0, 5%, 10%, 15%).

Sample	FWHM (222)	D (222) (Å)	AGS(Å)
$Cs_3Cu_2I_5$	0.40638	19.85413936	18.76603881
$Cs_3Cu_2I_5$@5%NaI	0.39844	20.24934543	20.1537243
$Cs_3Cu_2I_5$@10%NaI	0.38017	28.09612	26.314175
$Cs_3Cu_2I_5$@15%NaI	0.44485	18.13715808	19.13328409

The crystallite size(D) of a material can be calculated by the following Scherrer equation for the line broadening of the peak.

$$D = {}^{K\lambda}/_{\beta cos\theta} \qquad (1)$$

K is the Scherrer constant, which denotes the shape of the particle and is taken as 0.89 here; λ is wavelength of the X-ray beam used 0.15406 nm; β is the Full width at half maximum (FWHM) of the peak; θ is the Bragg angle. In this study, we conducted an analysis of the X-ray diffraction (XRD) pattern corresponding to the (222) lattice plane. The average grain size (AGS) of NCs can be determined by the calculated crystallite sizes of different crystal planes. As the concentration of NaI increases, FWHM of $Cs_3Cu_2I_5$ nanocrystals reaches a minimum at

10% NaI doping. Furthermore, the average grain size initially increases and then decreases with increasing NaI concentration. The maximal average grain size is observed at 10% NaI doping.

Table S2 summarizes some data on $Cs_3Cu_2I_5$ NCs since 2019. The NCs synthesized using the room temperature antisolvent crystallization method are superior in terms of monochromaticity, quantum yields and experimental conditions.

Table S2 Summary of NCs properties

Morphology	Method	Emission peak(nm)	PLQY (%)	FWHM（nm）	Temperature (℃)	Ref.
NCs	Hot injection	441	67	82	70	[2]
NCs	Hot injection	445	29.2	--	120	[3]
NCs	Modified hot injection	445	73.7	80	180	[4]
NCs	Modified hot injection	445	87	63	160	[5]
NCs	Antisolvent recrystallization	443	30	82	RT	[6]
NCs	Hot injection	445	90	87	160	[7]
NCs	Hot injection	441	~100	94	130-210	[8]
NCs	Hot injection	443	79.7	74.3	70	[9]
NCs	Dual-source co-evaporation	445	50.62	80	--	[10]
NCs	Hot injection	440	92.8	73	120	[11]
NCs	Antisolvent recrystallization	450	**90.2**	75	RT	**This work**

Table S3. PL lifetime of NCs (λ_{ex}=290 nm, λ_{em}=450 nm).

Sample	τ_1(ns)	τ_2(ns)	τ_{ave}(ns)
$Cs_3Cu_2I_5$	1262	6113	1267
10%NaI-$Cs_3Cu_2I_5$	1466	7413	1480

$$\tau_{average}=(A_1\tau_1^2+A_2\tau_2^2)/(A_1\tau_1 + A_2\tau_2) \quad (2)$$

Here, A_1 and A_2 standing for the weighting parameters, with τ_1 and τ_2 obtained from Equation 2. The average lifetimes for $Cs_3Cu_2I_5$ and $Cs_3Cu_2I_5$@10%NaI were determined to be 1267 ns and 1480 ns, respectively.

Reference

[1] T. Jiang, J. Wang, L. Xie, C. Bai, M. Wang, Y. Wu, F. Zhang, Y. Zhao, B. Chen, Y. Wang, In situ fabrication of lead-free $Cs_3Cu_2I_5$ nanostructures embedded in poly (vinylidene fluoride) electrospun fibers for polarized emission, ACS Applied Nano Materials, 5 (2022) 508-516 https://doi.org/https://doi.org/10.1021/acsanm.1c03323.

[2] P. Cheng, L. Sun, L. Feng, S. Yang, Y. Yang, D. Zheng, Y. Zhao, Y. Sang, R. Zhang, D. Wei, W. Deng, K. Han, Colloidal Synthesis and Optical Properties of All-Inorganic Low-Dimensional Cesium Copper Halide Nanocrystals, Angewandte Chemie-International Edition, 58 (2019) 16087-16091 https://doi.org/10.1002/anie.201909129.

[3] Z. Luo, Q. Li, L. Zhang, X. Wu, L. Tan, C. Zou, Y. Liu, Z. Quan, 0D $Cs_3Cu_2X_5$ (X = I, Br, and Cl) Nanocrystals: Colloidal Syntheses and Optical Properties, Small, 16 (2020) 1905226 https://doi.org/10.1002/smll.201905226.

[4] L. Lian, M. Zheng, W. Zhang, L. Yin, X. Du, P. Zhang, X. Zhang, J. Gao, D. Zhang, L. Gao, G. Niu, H. Song, R. Chen, X. Lan, J. Tang, J. Zhang, Efficient and Reabsorption-Free Radioluminescence in $Cs_3Cu_2I_5$ Nanocrystals with Self-Trapped Excitons, Advanced Science, 7 (2020) 2000195 https://doi.org/10.1002/advs.202000195.

[5] L. Wang, Z. Shi, Z. Ma, D. Yang, F. Zhang, X. Ji, M. Wang, X. Chen, G. Na, S. Chen, D. Wu, Y. Zhang, X. Li, L. Zhang, C. Shan, Colloidal Synthesis of Ternary Copper Halide Nanocrystals for High-Efficiency Deep-Blue Light-Emitting Diodes with a Half-Lifetime above 100 h, Nano Letters, 20 (2020) 3568-3576 https://doi.org/10.1021/acs.nanolett.0c00513.

[6] Y. Li, P. Vashishtha, Z. Zhou, Z. Li, S.B. Shivarudraiah, C. Ma, J. Liu, K.S. Wong, H. Su, J.E. Halpert, Room Temperature Synthesis of Stable, Printable $Cs_3Cu_2X_5$ (X = I, Br/I, Br, Br/Cl, Cl) Colloidal Nanocrystals

with Near-Unity Quantum Yield Green Emitters (X = Cl), Chemistry of Materials, 32 (2020) 5515-5524 https://doi.org/10.1021/acs.chemmater.0c00280.

[7] F. Zhang, W. Liang, L. Wang, Z. Ma, X. Ji, M. Wang, Y. Wang, X. Chen, D. Wu, X. Li, Y. Zhang, C. Shan, Z. Shi, Moisture-Induced Reversible Phase Conversion of Cesium Copper Iodine Nanocrystals Enables Advanced Anti-Counterfeiting, Advanced Functional Materials, 31 (2021) 2105771 https://doi.org/10.1002/adfm.202105771.

[8] S. Liu, H. Liu, G. Zhou, X. Li, S. Wang, Water-induced crystal phase transformation of stable lead-free Cu-based perovskite nanocrystals prepared by one-pot method, Chemical Engineering Journal, 427 (2022) 131430 https://doi.org/10.1016/j.cej.2021.131430.

[9] Y.-H. Cheng, R. Suzuki, N. Shinotsuka, H. Ebe, N. Oshita, R. Yamakado, T. Chiba, A. Masuhara, J. Kido, Gel permeation chromatography process for highly oriented $Cs_3Cu_2I_5$ nanocrystal film, Scientific Reports, 12 (2022) 4620 https://doi.org/10.1038/s41598-022-08760-6

[10] X. Zhao, A. Luo, Z. Lin, X. Zhong, Z. Zhu, Q. Lin, H. Su, Lead-free $Cs_3Cu_2I_5$ perovskite vertical cavity surface emitting lasers with low threshold, Journal of Luminescence, 253 (2023) 119434 https://doi.org/10.1016/j.jlumin.2022.119434.

[11] K. Qu, Y. Lu, P. Ran, K. Wang, N. Zhang, K. Xia, H. Zhang, X. Pi, H. Hu, Y. Yang, Zn (II)-Doped Cesium Copper Halide Nanocrystals with High Quantum Yield and Colloidal Stability for High-Resolution X-Ray Imaging, Advanced Optical Materials, 11 (2023) 2202883 https://doi.org/https://doi.org/10.1002/adom.202202883.
